# The Influence of Bariatric Surgery on Pregnancy and Perinatal Outcomes—A Case-Control Study

**DOI:** 10.3390/jcm9051324

**Published:** 2020-05-02

**Authors:** Anna Różańska-Walędziak, Maciej Walędziak, Paweł Bartnik, Joanna Kacperczyk-Bartnik, Michał Janik, Piotr Kowalewski, Andrzej Kwiatkowski, Krzysztof Czajkowski

**Affiliations:** 1 2nd Department of Obstetrics and Gynecology, Medical University of Warsaw, Karowa 2 St., 00-315 Warsaw, Poland; aniaroza@tlen.pl (A.R.-W.); bartnik.pawel@gmail.com (P.B.); asiakacperczyk@gmail.com (J.K.-B.); krzysztof.czajkowski@wum.edu.pl (K.C.); 2 Department of General, Oncological, Metabolic and Thoracic Surgery, Military Institute of Medicine, Szaserów 128 St., 04-141 Warsaw, Poland; mjanik@wim.mil.pl (M.J.); pkowalewski@wim.mil.pl (P.K.); akwiatkowski@wim.mil.pl (A.K.)

**Keywords:** bariatric surgery, pregnancy, gestational diabetes mellitus, pregnancy-induced hypertension

## Abstract

Introduction: Obesity in pregnant women increases the incidence of pregnancy-induced comorbidities and the rate of operative deliveries. Purpose of the Study: As bariatric surgery is the reference method of treatment of obesity, we wanted to evaluate its influence on the course of pregnancy and perinatal outcomes. Material and Methods: Data was collected from 627 female patients after bariatric surgery, of whom 107 had a history of pregnancy after the surgery, and 345 non-bariatric patients who had a delivery at a tertiary perinatal center. Sixty-one cases were matched (1:1) with controls for age, pre-pregnancy BMI and presence of pre-pregnancy comorbidities. The main endpoints were gestational diabetes mellitus (GDM), pregnancy-induced hypertension (PIH), small (SGA) and large for gestational age infants (LGA) and cesarean sections (CS). Results: Patients after bariatric procedures were significantly less likely to have GDM (19.67%/37.7%; *p* = 0.0433), PIH (11.47%/16.39%; *p* = 0.6072) and preterm delivery (13.11%/37.7%; *p* = 0.0026). The CS rate was higher (57.38%/40.98%; *p* = 0.0987). There was an increased risk of SGA (18.03%/13.11%; *p* = 0.6072) and a decreased risk of LGA (6.56%/16.39%; *p* = 0.146). Conclusions: Patients after bariatric surgery have a decreased risk of pregnancy-induced comorbidities, preterm deliveries and LGA infants, with an increase in rate of CS and SGA infants compared to general population matched for pre-pregnancy BMI, age and presence of pre-pregnancy comorbidities.

## 1. Introduction

Obesity is a major healthcare problem with an increasing global prevalence, having reached 650 million people in 2016, which represents 13% of the world population [1]. Obesity is a well-known risk factor for many concomitant diseases, distinctively diabetes mellitus type 2, hypertension, heart disease and obstructive sleep apnea syndrome (OSAS). It can also lead to sexual dysfunctions in both sexes. Obese women of reproductive age can suffer from infertility due to hormonally related ovulation dysfunction. Obesity in pregnant women increases the incidence of gestational diabetes mellitus (GDM), pregnancy-induced hypertension (PIH), which also influences the way of delivery, with an increase in number of vacuum deliveries and cesarean sections [2,3]. Bariatric surgery is the reference method of treatment for obesity, because of the durability and pace of the weight loss, reduction of symptoms and remission of concomitant diseases. As the prevalence of obesity in women of reproductive age is rising every year, the number of pregnancies after bariatric surgery is also increasing, creating a demand for knowledge among obstetricians about the influence of the bariatric surgery on the course of pregnancy. Women constitute up to 80% of bariatric patients, most of them being of reproductive age [4,5,6]. Bariatric surgery leads to various micronutrient deficiencies in pregnant women, with possible influence on fetal development, though further studies are needed on the subject [3,7,8,9]. Bariatric surgery and the resulting weight loss, reduction in adipose tissue as well as alteration of the gastrointestinal absorption lead to hormonal and metabolic changes that may affect the well-being of the woman and the fetus. They can also influence the incidence and course of pregnancy-induced co-morbidities. Bariatric surgery is known to have influence on the pregnancy and neonatal outcomes, increasing the rate of small for gestational age (SGA) infants and maternal anemia and decreasing the risk of GDM, PIH and large for gestational age infants (LGA) infants [10,11,12,13].

## 2. Purpose of the Study

In our study, we analyzed a group of patients with a history of pregnancy after bariatric surgery and compared them with a matched group of controls to examine the influence of restrictive and malabsorptive procedures on the course of pregnancy, pregnancy-induced diseases and perinatal outcomes. The study was designed as a retrospective case-control. The patients in the study group had a history of previous bariatric surgery, the mean time-to-conception interval after the surgery was 23.25 (± 16.9) months. The control group included patients without a history of bariatric surgery, matched for: age, BMI at the beginning of the pregnancy, presence of pre-pregnancy diabetes mellitus type 2 (PGDM) and pre-pregnancy hypertension (PPH). The primary end-points of our study were the occurrence of GDM, PIH, intrauterinal growth restriction (IUGR), small for gestational age (SGA) and large for gestational age (LGA) infants, neonatal intensive care unit (NICU) admission as well as premature deliveries, vacuum deliveries (VE) and cesarean sections (CS). We also analyzed the gestational weight gain (GWG) and the body mass index (BMI) at the moment of the delivery.

## 3. Material and Method

### 3.1. Participants—Subjects

We collected data from 627 female patients with a history of bariatric surgery, using paper and internet original survey. The recruitment was based on the bariatric center register and cooperation with Polish Bariatric Patients Society. There were 107 patients with a history of at least one pregnancy after bariatric surgery, out of whom 74 patients had laparoscopic sleeve gastrectomy (LSG), 21 laparoscopic Roux-en-Y gastric bypass (LRYGB), 9 adjustable silicone gastric banding (ASGB) and 3 other bariatric procedures. There were 77 patients from the group who met the inclusion criteria—having had at least one pregnancy ended with a delivery of a single live-born neonate after the bariatric surgery. The exclusion criteria were: multiple pregnancies, stillbirth and no sufficient data for the matching process. They were further surveyed for the data about the course of the pregnancy, maternal and neonatal outcomes. The vast majority of patients had spontaneous pregnancies, only one patient became pregnant after using methods of reproductive assistance. The approval from Military Institute of Medicine Ethics Committee was obtained on 22^nd^ August 2018 (no. 117/WIM/2018).

### 3.2. Participants—Controls

We analyzed pregnancy and perinatal outcomes of 345 patients who were hospitalized and had a delivery at a tertiary perinatal center. The preliminary collected data included patients’ age, BMI at the beginning of the pregnancy, PGDM and PPH. They were further surveyed for data about the course of the pregnancy, and maternal and neonatal outcomes.

### 3.3. Matching

Propensity scores (i.e., the estimated probability of undergoing bariatric surgery before pregnancy on the basis of the preoperative values) were estimated for each patient by use of logistic regression. Potential confounding variables were considered and included age, body mass index, hypertension and diabetes type 2. We used a greedy matching algorithm (developed by Mayo Clinic group—http://bioinformaticstools.mayo.edu/research/gmatch/) to match each case to a control, restricting successful matches to those for whom the propensity scores did not differ by more than 0.2 units. Balance on potential confounding variables between the matched controls and cases were evaluated with standard univariable summary statistics and absolute standardized difference scores (absolute value of difference in means or proportions, divided by a combined estimate of standard deviation among the groups being compared). Variables with an absolute standardized difference score of less than 0.10 were considered adequately balanced. McNemar test was used to assess effect of exposure on dichotomous outcomes. The Cochran–Mantel–Haenszel test was used in the analysis of matched data. Continuous outcomes were assessed using the Wilcoxon signed-rank test.

### 3.4. Study Design

The study group and the control group were compared for the following variables: occurrence of GDM and PIH, premature delivery (defined as delivery before 37th week of pregnancy), method of delivery (vaginal spontaneous delivery, vacuum delivery, scheduled or urgent cesarean section due to pathological cardiotocography tracings or other indications), pregnancy duration and neonatal data, with strong emphasis on SGA and LGA neonates. Neonatal data included birth weight, birth length, ponderal index and Apgar score. SGA was defined as below the 10th percentile and LGA as over 90th percentile using population adjusted birth weight scores.

Pregnancy-induced hypertension was defined as de novo onset of hypertension (>140 mmHg systolic or >90 mmHg diastolic) after 20th week of gestation. Gestational diabetes mellitus was diagnosed with the oral glucose tolerance test with 75 g glucose (OGTT), administered after 8 h of fasting. GDM was identified in case of fasting blood glucose level of ≥5.1 mmol/L up to 6.9 mmol/L, ≥10 mmol/L after first hour of glucose administration and/or ≥ 8.5 mmol/L up to 11.0 mmol/L after the second hour. OGTT was recommended between 24th and 28th week of gestation in the general population and in the 1st trimester in case of BMI ≥ 30 kg/m^2^, history of GDM in previous pregnancies, family history of diabetes mellitus type 2 and having given birth to a child of ≥4500 g birth weight [14]. Though our national guidelines suggest an alternative form of screening—home glucose monitoring—for patients after bariatric surgery with dumping syndrome, the vast majority patients included in the study had OGTT administered [15].

## 4. Results

Sixty-one pairs were created, matched for age, BMI at the beginning of pregnancy and presence of pre-pregnancy comorbidities, such as diabetes mellitus and hypertension. Standardized differences in the propensity score matched sample are shown in Table 1 and demographic characteristics in Table 2.

The general characteristics about the pregnancy length and neonatal data is presented in Table 3.

The mean pregnancy duration was 39 weeks in the study group and 37 weeks in the control group. Patients who had undergone bariatric surgery before the pregnancy had longer pregnancies, the difference being statistically significant (*p* < 0.0001). The Apgar score (5th minute after the delivery) used to evaluate neonates’ condition was higher in the study group than in the control group (*p* = 0.0027) with a statistically significant difference present in the distribution of the results.

Statistically significant differences were observed neither in the neonates’ birth weight nor in the birth length between the study group and the control group. The ponderal index was slightly lower in the study group, although the difference observed was not statistically significant.

We did not observe either maternal nor neonatal fatal cases in either study or control groups.

Patients after bariatric procedures were significantly less likely to have GDM than the control group (19.67% vs. 37.7%; *p* = 0.0433). Preterm delivery was less likely in the bariatric group than in the control group (13.11% vs. 37.7%; *p* = 0.0026). The differences found in the incidence of GDM and premature delivery were both statistically significant.

The incidence of pregnancy-induced hypertension was lower in the study group; however, it was not observed to be of statistical significance (11.47% vs. 16.39%; *p* = 0.6072).

Patients after bariatric procedures were less likely to have a vaginal spontaneous delivery (40.98% vs. 59.02%; *p* = 0.0708). Forceps and vacuum delivery were observed in only two cases in the study group (3.28%).

The proportion of CS was higher in the bariatric group than in the control group (57.38% vs. 40.98%; *p* = 0.0987). Patients after bariatric procedures most often had scheduled CS, followed by urgent CTG indications and other urgent indications (22 vs. 10 vs. 3).

Neonates in the bariatric group were more likely to be classified to be SGA and less likely to be LGA (SGA—18.03% vs. 13.11%; *p* = 0.6072/LGA—6.56% vs. 16.39%; *p* = 0.146).

The results are summarized in Table 4.

## 5. Discussion

Our principal findings indicate lower incidence of GDM and PIH in patients after bariatric surgery when compared to general population matched for pre-pregnancy BMI, age and presence of pre-pregnancy comorbidities. Similar to the literature, we found a lower rate of preterm deliveries and LGA infants with an increase in rate of SGA infants in the bariatric surgery group. We also observed an increase in number of scheduled CS with a decrease in proportion of urgent CS in bariatric patients.

Compared to other studies, our analysis includes a higher number of patients, making it possible to present results of statistical significance. The uniqueness of our study is the comparison between patients after bariatric surgery and patients matched for BMI at the beginning of pregnancy, age and pre-pregnancy co-morbidities. Most case-control studies about the influence of bariatric surgery on the course of pregnancy and neonatal outcomes include controls matched for BMI from before the operation and often lack information about pre-pregnancy co-morbidities. This makes it possible to see an increased positive impact of bariatric surgery on the pregnancy adverse outcomes, but also leads to interpretation bias, as obese patients in the control group are more likely to have pregnancy complications due to obesity. This emphasizes the positive influence of bariatric surgery on reduction of obesity-related complications, but excludes the possibility of comparison of bariatric patients with general population. In our study, we compare patients after bariatric surgery to patients from the general population, not only those who are obese. Pre-existing hypertension can strongly influence the birth weight of the neonate and further the evaluation of the correlation between bariatric surgery and the risk of LGA and IUGR. Pre-pregnancy diabetes mellitus, especially with poor control of blood glucose levels can lead to LGA and also influence the results of the analysis. Both PPH and PGDM may cause the necessity of ending the pregnancy preterm and can lead to pregnancy and neonatal complications, non-attributable to bariatric surgery, hence the importance of including them in the matching process.

Johansson et al. presented a study based on the Swedish Birth Register, in which they matched patients after bariatric surgery and controls for the pre-surgery BMI, age, parity, smoking and educational level [16]. They observed a reduction in the incidence of GDM (1.9% in the study group vs. 6.8% in the control group) and LGA (8.4% vs. 22.4%). There was an increased risk of SGA in the bariatric group—15.6% vs. 7.6% and a reduction in the pregnancy length—273.0 days vs. 277.5 days. We matched the patients for BMI at the beginning of pregnancy, which may be the reason for the differences in the results. In our study, 19.67% patients after bariatric procedures were diagnosed with GDM compared with 37.7% in the study group. The higher incidence of GDM may be result of population disparity, but also of different GDM diagnosis criteria. We did not observe a reduction in the pregnancy length in patients after bariatric surgery and furthermore, preterm delivery was less likely in the bariatric group than in the control group—13.11% vs. 37.7%. Our analysis presented a higher proportion of SGA among neonates of bariatric patients, and lower proportion of LGA neonates. A major limitation of the Johansson’s study was that 98% of bariatric procedures were gastric bypass surgery. The increase in the rate of SGA infants after bariatric surgery has been widely discussed in the literature, with some studies indicating that the increase is comparable in pregnancies after SG to those after GB [10,13,17], while others suggest that the increase is mostly observed after LRYBG [8], or even determine no increase in the rate of SGA [18].

Aricha-Tamir et al. analyzed a group of patients with a history of delivery before and after bariatric surgery [19]. The study mostly included patients after LSG and vertical banded gastroplasty (VGB) with only 3.8% patients after LRYGB. The study presented a decrease in proportion of hypertension (without determining whether pre-pregnancy or pregnancy-induced hypertension) after the surgery from 31.9% to 16.7% and of GDM—from 19.3% to 3.5%. The analysis presented an increase in the number of CS, from 24.3% to 31.9%, with a decrease in urgent CS of 5%. Most studies present an increase in the rate of CS after bariatric surgery [12,20], although some researchers observed the opposite [2]. In our study, we also observed an increase in number of scheduled CS with a decrease in proportion of urgent CS. The difference between pre-surgery and after-surgery pregnancies in Aricha-Tamir’s study was not statistically significant in the proportion of LGA neonates or the birth weight as general.

Galazis et al. presented a meta-analysis of the perinatal outcomes after bariatric surgery, based on 17 studies [21]. The primary endpoints were preeclampsia, GDM, maternal anemia, premature delivery, LGA and SGA neonates, NICU admission and perinatal mortality. The risk of preeclampsia, GDM and LGA neonates was 50% lower after bariatric surgery, unlike the rate of SGA neonates, which was 80% higher and of premature delivery—28% higher. The analysis did not reveal any differences in the proportion of CS. The results obtained in our study were comparable in terms of GDM. We cannot compare the data about hypertension, as we included all cases of PIH and Galazis et al. analyzed only cases of preeclampsia (PIH followed by additional proteinuria or other maternal organ dysfunction). In our study, we observed a lower rate of premature deliveries in the bariatric group than in the control group.

A more than three-fold decrease in the incidence of GDM was observed in a study by Burke et al., who compared a group of women with a delivery before and after bariatric surgery, having based on a private insurance claims database [22]. Kwong et al. presented a meta-analysis about the influence of bariatric surgery on pregnancy course and perinatal outcomes. The analysis showed that bariatric surgery led to reduced rates of GDM (OR 0.20), LGA infants (OR 0.32) and all hypertensive disorders (OR 0.38) and an increase in SGA infants (OR 2.16) and preterm deliveries (OR 1.35) [20]. The meta-analysis included studies comparing bariatric patients with control subjects matched for pre-surgery BMI. In our study, we compared bariatric patients matched with controls for BMI at the beginning of the pregnancy, age, presence of PGDM and PPH. The impact of bariatric procedures on the reduction of the rate of GDM and LGA, together with an increased rate of SGA infants was also observed in a case-control study by Chevrot et al. [23].

The question about the incidence of GDM in pregnant patients after bariatric surgery is whether the result of OGTT, performed with liquid glucose solution is reliable and whereas the absorption changes after the operation do or do not influence blood sugar results and therefore the result of OGTT. A reduction in the glucose absorption might lead to lower blood sugar levels after the glucose intake, so it remains to be analyzed whether the real proportion of GDM is the one found with OGTT screening. Home glucose monitoring is more reliable and is advised in the guidelines.

Home glucose monitoring with evaluation of fasting and postprandial blood sugar levels is suggested in our national guidelines as an alternative form of GDM screening in patients after bariatric surgery with dumping syndrome [15]. The American College of Obstetricians and Gynecologists recommends home glucose monitoring with evaluation of fasting blood glucose levels and two hours postprandial, whereas our recommendations suggest after one hour [24]. However, the differences between countries in diagnostic algorithms and blood glucose thresholds lead to heterogeneity in study results [25]. The suggested period of monitoring in both guidelines is of one week between 24th and 28th week of gestation [26], which is repetitively emphasized in other studies and recommendations [4,27,28]. Even though the recommendations remain clear, the vast majority patients included in the study admitted having had OGTT administered. The problem of introduction and adherence to the guidelines remains to be ameliorated. Rottenstreich emphasizes the problem of hypoglycemia after OGTT in bariatric patients as it affects almost 50% of pregnant patients after bariatric surgery with incidence of 83% in patients after LRYGB [29]. Hypoglycemia in OGTT is correlated with a higher proportion of SGA infants and patients after bariatric surgery may experience hypoglycemia during a substantial portion of time, which may be the reason for the increased risk of IUGR and SGA.

## 6. Limitations of the Study

The main limitation of our study is its retrospective nature and the possibility of recall and selection bias. Having collected data with a paper and internet survey resulted in the impossibility of obtaining all the necessary data about all pregnant women and their newborns. Additionally, there is a possibility of sample bias, as patients from the control group were hospitalized in a 3rd degree perinatal center and the percentage of patients with high risk pregnancies was higher than in general population.

## 7. Conclusions

Bariatric surgery is well known to reduce the risk of GDM, PIH, LGA infants, with an increase in the number of SGA infants. Most studies compare pregnancy complications and neonatal outcomes in bariatric patients with obese control groups, matched for preoperational BMI. As obesity is an independent risk factor of many comorbidities, those studies mostly show the positive influence of bariatric procedures and the following reduction of body weight on reduction of obesity-dependent pregnancy complications and neonatal outcomes. In our study, we presented a comparison of patients after bariatric procedures with a group of controls matched for BMI at the beginning of pregnancy. Our results show that bariatric patients have a decreased risk of pregnancy comorbidities and LGA even when compared with non-obese population. Additionally, we included in our matching presence of pre-pregnancy diabetes mellitus and hypertension, to exclude their influence on incidence of LGA, SGA and perinatal complications. 

Although all types of bariatric operations are meant to lead to a similar therapeutic effect, their influence on the physiology of digestion and absorption of nutrients is based on different mechanisms. We acknowledge the importance of heterogeneity of bariatric procedures and the need for comparing the influence of restrictive and malabsorptive procedures on the pregnancy and neonatal adverse outcomes, which will be subject of our further studies. 

## Figures and Tables

**Table 1:** Standardized differences in propensity score.

	d
Age	0.070
Body Mass Index	0.088
Diabetes	0.063
Hypertension	0.047

d: standardized differences.

**Table 2:** Demographic characteristics.

Characteristic	Original Cohort			Matched Cohort		
	Cases	Controls	*p*-Value	Cases	Controls	*p*-Value
	*N* = 77	*N* = 345		*N* = 61	*N* = 61	
	Mean (SD) or %			Mean (SD) or %		
Age (years)	34.92 (± 5.77)	30.76 (± 4.3)	<0.001	34.03 (± 5.56)	33.69 (± 4.19)	0.783
BMI (kg/m2)	29.27 (± 5.65)	23.86 (±5.05)	<0.001	28.17 (± 4.15)	28.63 (± 6.11)	0.822
Pre-Op Hypertension	11.69%	13.04%	0.852	13.11%	14.75%	1.000
Pre-Op Diabetes Mellitus	6.49%	16.81%	0.022	6.56%	8.2%	1.000

BMI: Body Mass Index.

**Table 3:** Pregnancy length and neonatal data.

	Cases (Median (Q_1–_Q_3_))	Controls (Median (Q_1–_Q_3_))	*p*-Value
5th minute Apgar	10 (10–10)	10 (9–10)	0.0027
Birth weight (g)	3200 (2860–3550)	3140 (2830–3540)	0.9495
Birth length (cm)	54 (52–56)	54 (50–55)	0.2057
Ponderal index (kg/cm^3^) (1000 × weight/(length × length × length))	19.91 (18.51–22.99)	20.64 (19.06–21.52)	0.3695
Pregnancy duration (days)	273 (266–280)	259 (255–269)	0.0002
Week of gestation at the delivery (weeks)	39 (38–40)	37 (36–38)	<0.0001

**Table 4:** Influence of bariatric surgery on pregnancy.

Characteristics	Cases	Controls	*p*-Value
gestational diabetes mellitus	19.67%	37.7%	0.0433
preterm delivery	13.11%	37.7%	0.0026
pregnancy-induced hypertension	11.47%	16.39%	0.6072
vaginal spontaneous delivery	40.98%	59.02%	0.0708
forceps or vacuum delivery	3.28%	0	
cesarean section	57.38%	40.98%	0.0987
small for gestational	18.03%	13.11%	0.6072
large for gestational	6.56%	16.39%	0.146

## Abbreviations

OSAS	obstructive sleep apnea syndrome
GDM	pregnancy diabetes mellitus
PIH	pregnancy-induced hypertension
PGDM	pre-pregnancy diabetes mellitus type 2
PPH	pre-pregnancy hypertension
IUGR	intrauterine fetal growth restriction
SGA	small for gestational age
LGA	large for gestational age
NICU	neonatal intensive unit
VE	vacuum extractor
CS	cesarean section
GWG	gestational weight gain
BMI	body mass index
LSG	laparoscopic sleeve gastrectomy
LRYGB	laparoscopic Roux-en-Y gastric bypass
ASGB	adjustable silicone gastric banding
LGB	laparoscopic gastric banding
OGTT	oral glucose tolerance test
